# Fungal *Cordyceps* Nucleosides and Analogs as Potential Anti-Glioblastoma PD-L1 Inhibitors: An In Silico Multiparameter Optimization (MPO) Design

**DOI:** 10.3390/ijms27115024

**Published:** 2026-06-02

**Authors:** Felipe Muñoz-González, Martiniano Bello, José Correa-Basurto, Cindy Bandala

**Affiliations:** 1 Laboratorio de Diseño y Desarrollo de Nuevos Fármacos e Innovación Biotecnológica, SEPI-ESM, Instituto Politécnico Nacional, Mexico City 11340, Mexico; felipemgonzalez1@yahoo.com.mx (F.M.-G.);; 2 Laboratorio de Neurociencia Traslacional, Escuela Superior de Medicina, Instituto Politécnico Nacional, México City 11340, Mexico

**Keywords:** glioblastoma, cancer, nucleosides, cordycepin

## Abstract

Immune checkpoint modulation has emerged as a promising strategy in cancer therapy, including the treatment of aggressive tumors such as glioblastoma. Among these targets, programmed death-ligand 1 (PD-L1) plays a key role in tumor immune evasion and represents an attractive target for small-molecule inhibitor development. In this study, a virtual screening approach was applied to identify potential PD-L1 modulators within a library of nucleoside-related compounds and structurally similar molecules. A dataset of 400 compounds was evaluated using molecular docking to predict their binding affinity (free energy values and binding pose) toward PD-L1. The resulting complexes were analyzed to identify nonbond interactions within the hydrophobic pocket formed at the PD-L1 dimer interface. In addition to docking results, physicochemical descriptors associated with drug-likeness and blood-brain barrier penetration were calculated, including lipophilicity, molecular weight, hydrogen bond donors and acceptors, as well as topological polar surface area. To integrate these parameters, a multiparameter optimization (MPO) score was implemented. Finally, molecular dynamics simulations of protein-ligand interactions were performed to explore the structural stability for 100 ns using the most promising ligands. The analysis revealed that several top-ranked compounds exhibited favorable docking scores and physicochemical properties compatible with drug-like behavior. Interestingly, BMS-1, a known PD-L1 inhibitor, was identified among the highest-scoring compounds, supporting the reliability of the MPO protocol. Furthermore, multiple candidates displaying nucleoside-like scaffolds combined with reduced polarity and moderate lipophilicity emerged as promising molecules according to the MPO ranking. Overall, the results suggest that nucleoside-derived scaffolds may represent a viable starting point for the development of small-molecule PD-L1 modulators with potential applicability in glioblastoma therapy.

## 1. Introduction

Glioblastoma is the most aggressive primary brain tumor in adults and is characterized by rapid progression, high invasiveness, and poor overall survival despite current therapeutic strategies [1]. Standard treatment includes surgical resection followed by radiotherapy and chemotherapy, most commonly with temozolomide; however, these approaches provide only limited clinical benefit, with median survival remaining approximately 15 months [2]. The failure of conventional therapies is largely attributed to tumor heterogeneity, invasive growth, and the presence of a highly immunosuppressive tumor microenvironment [3].

Among the mechanisms contributing to immune evasion, PD-L1 plays a central role. PD-L1 is frequently overexpressed in glioblastoma and interacts with the PD-1 receptor on cytotoxic T lymphocytes, leading to T-cell exhaustion and impaired antitumor immune responses [4]. Elevated PD-L1 expression has been associated with higher tumor grade and poor patient prognosis [5], highlighting its relevance as both a prognostic biomarker and a therapeutic target [6]. Although immune checkpoint inhibitors have shown promise in several malignancies, their efficacy in glioblastoma remains limited, partly due to restricted drug delivery to the tumor site and the unique immunological environment of the brain [7].

A major challenge in the treatment of glioblastoma is the presence of the blood–brain barrier (BBB), which severely restricts the penetration of many therapeutic agents into the central nervous system [8]. Although partial disruption of the BBB can occur in tumor regions, this effect is highly heterogeneous and does not ensure uniform drug distribution. Furthermore, infiltrative tumor cells often reside in areas where the BBB remains intact, preventing adequate exposure to many hydrophilic or high-polarity compounds [8]. It has been proposed that drug delivery in this context may depend on alternative mechanisms such as nucleoside transporter-mediated uptake or the use of targeted delivery systems, including nanoparticles, liposomes, or molecular conjugates [9,10,11]. In several cases, the observed antitumor effects may be associated with tumor-induced disruption of the barrier or experimental conditions designed to enhance drug exposure within the brain [12].

In this context, the discovery of small molecules capable of modulating immune checkpoint pathways while maintaining favorable pharmacokinetic properties represents an important unmet need. Structure-based drug design and virtual screening approaches have emerged as valuable tools for identifying compounds that can interact with key regions of the PD-L1 interface [13]. Previous computational studies combining molecular docking, pharmacophore modeling, and molecular dynamics (MD) simulations have successfully identified compounds capable of binding to key hotspots within the PD-L1 interface [14,15]. These studies demonstrate that computational screening strategies can efficiently prioritize potential PD-L1 inhibitors for further experimental validation.

**Figure 1:**
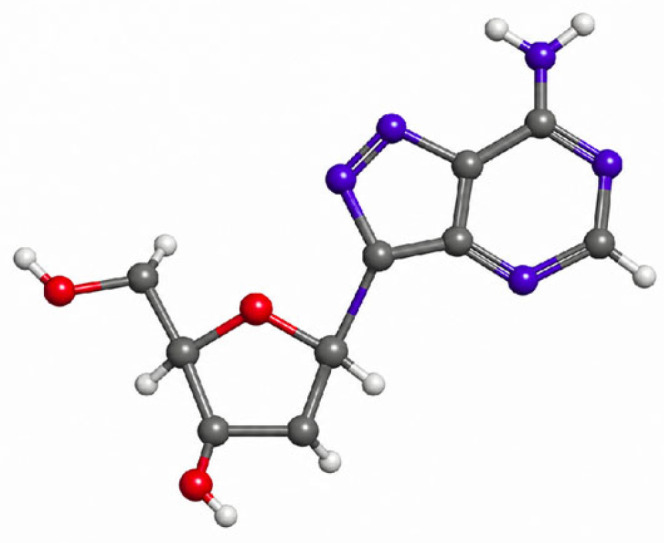
Cordycepin (3′-deoxyadenosine) 3D structure. Color code: oxygen: red, nitrogen: blue, carbon: gray, and hydrogen: white.

Natural nucleosides have attracted considerable attention as potential therapeutic scaffolds due to their diverse biological activities. Cordycepin (3′-deoxyadenosine), a natural nucleoside isolated mainly from fungi of the genus *Cordyceps*, has been reported as an anti-inflammatory and immunomodulatory compound in different cell types, including lungs, macrophages, chondrocytes, cardiac tissue, glia, and brain tissue [16,17], as well as exhibiting antitumor effects [18]. Anti-inflammatory and neuroprotective effects have also been described in the brains of mice affected by ischemia [19]. The chemical structure of cordycepin is shown in Figure 1.

In glioblastoma models, cordycepin has demonstrated the ability to inhibit cell proliferation, induce caspase-mediated apoptosis, cause cell cycle arrest, and reduce tumor migration and tissue invasion in human glioblastoma cell lines such as U87, U251, T98G, and LN-229 [20]. These effects have been associated with the modulation of oncogenic signaling pathways, including PI3K/AKT, MYC, MMPs, and epithelial–mesenchymal transition processes [21]. However, these findings are largely derived from in vitro conditions, where the compound has direct access to tumor cells without the constraints imposed by the BBB.

From a physicochemical perspective, cordycepin is a low molecular weight molecule (~251 Da) with high polarity, multiple hydrogen bond donors and acceptors, and an elevated topological polar surface area. These physicochemical characteristics are typically associated with limited passive diffusion across the BBB [22], which may restrict its clinical applicability in the treatment of central nervous system (CNS) tumors.

To overcome these limitations, structural modification of nucleoside scaffolds has been widely explored as a strategy to improve pharmacokinetic properties, including membrane permeability and metabolic stability. Several natural and synthetic nucleoside analogs, such as adenosine, tubercidine, and toyocamycin, have demonstrated antitumor and signaling modulatory properties, reinforcing the therapeutic potential of bioactive nucleosides derived from microorganisms as platforms for drug discovery [23,24,25]. Overall, available evidence indicates that cordycepin and its structural analogs exhibit antitumor activity against glioblastoma in cellular models [26]. Table 1 shows in vitro and in vivo studies of PD-L1 inhibitors that have shown antitumor activity.

However, their biological targets are not fully characterized at the 3D structural level, and their clinical application is limited by poor permeability across the BBB, which restricts effective drug concentrations in the CNS [32]. Consequently, the identification or design of structural derivatives with improved pharmacokinetic properties and BBB penetration represents a critical step toward the development of nucleoside-based therapeutics for glioblastoma.

Based on these considerations, the present study aims to identify and evaluate novel cordycepin-related compounds with improved drug-like properties and potential activity against PD-L1. Using an in silico approach combining molecular docking, MD simulations, and multiparametric optimization (MPO), we sought to prioritize candidate molecules with favorable binding affinity and physicochemical characteristics compatible with BBB penetration.

## 2. Results

### 2.1. Molecular Docking

Virtual screening of a 400-compound library revealed multiple molecules with favorable predicted binding affinities toward the PD-L1 target receptor. The docking protocol was first validated through a redocking procedure, which successfully reproduced the crystallographic binding pose with a root-mean-square deviation (RMSD) of 1.96 Å (Supplementary File S1) with the inhibitor VYC (PubChem 168433094, https://pubchem.ncbi.nlm.nih.gov/compound/168433094, accessed on 10 January 26), supporting the reliability of the computational strategy. To confirm poses, two other known PD-L1 ligands were used to validate the procedure, namely PDB (Protein Data Bank) structures 5J89 and 6NM8 (X-ray structures) (Table 2), both of which are reported to have in vitro inhibitory activities [33,34].

The predicted binding energies showed a broad distribution, indicating substantial variability in ligand–receptor complementarity across the screened chemical space. Notably, of the 400 compounds tested, several exhibited stronger predicted affinities than co-crystallized VYC, suggesting their potential ability to competitively occupy the binding pocket (Supplementary File S2).

**Figure 2:**
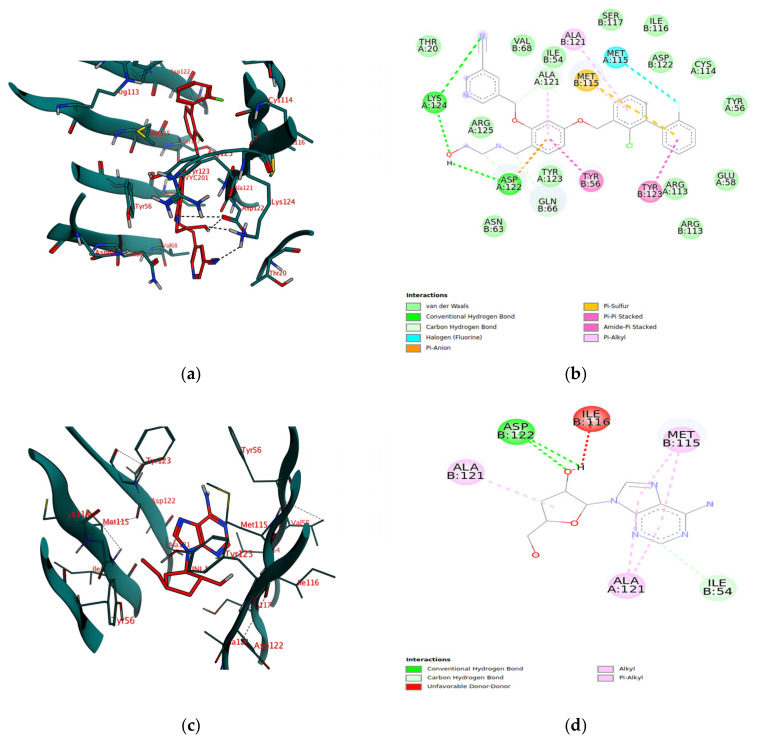
Binding pocket of cordycepin and VYC ligands in PD-L1 crystal (PDB ID: 8OR1): (**a**) 3D figure of VYC ligand present in 8OR1 PDB crystal; pose is obtained from redocking pose, doted lines represent hydrogen bonds; (**b**) 2D interaction map of VYC ligand with PD-L1, additional interactions are detected; (**c**) 3D interaction figure of cordycepin with PD-L1, interactions stabilizes de dimer; (**d**) 2D interaction map of cordycepin interaction with PD-L1; three hydrogen bonds are formed for both ligands and weak interactions.

Top-ranked candidates were prioritized based on binding affinity and subsequently inspected to characterize their interaction patterns. Structural analysis revealed recurrent non-bond interactions with key residues (Ile116-Tyr123) located within the active site, highlighting a conserved interaction network likely contributing to ligand stabilization (Figure 2). Key predicted protein–ligand interactions for the proposed lead compounds are summarized in Table 3. The binding energies ranged from −10.8 to −7.0 kcal/mol. The proposed lead compounds demonstrated several hydrogen bond interactions, hydrophobic contacts, and π-related interactions with residues implicated in maintaining the structural integrity of the binding pocket. These interaction profiles support the hypothesis that the selected molecules possess structural features compatible with effective receptor engagement.

### 2.2. Molecular Dynamics Simulations

#### 2.2.1. Equilibration of the System Through MD Simulations

**Figure 3:**
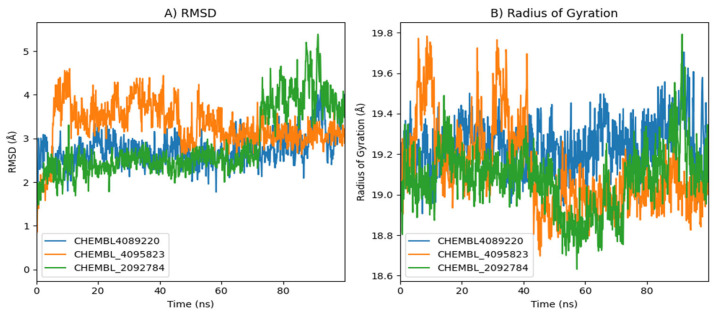
Molecular dynamics analysis of the protein–ligand complexes for CHEMBL4089220, CHEMBL_4095823, and CHEMBL_2092784: (**A**) RMSD of the protein backbone over 100 ns of simulation, showing the structural stability of each complex. (**B**) Radius of gyration (Rg) as a function of time, reflecting the overall compactness of the protein during the simulation.

The RMSD and radius of gyration (Rg) analyses provide complementary insights into the structural stability and compactness of the systems throughout the simulations. RMSD profiles indicate that all systems reach a relatively stable regime after an initial equilibration phase, although with distinct behaviors: the CHEMBL4089220–PD-L1 complex remains consistently stable with moderate fluctuations (~2.5–3.2 Å), suggesting a well-equilibrated structure; the CHEMBL_4095823–PD-L1 complex exhibits higher initial deviations (~4–4.5 Å) followed by gradual stabilization near ~3 Å, indicative of an early conformational rearrangement, while the CHEMBL_2092784–PD-L1 complex shows increased fluctuations and a progressive rise up to ~5 Å, pointing to enhanced structural flexibility or a possible conformational transition (Figure 3). In contrast, Rg values remain within a narrow range (~18.8–19.6 Å) across all systems, indicating that the overall compactness of the protein is largely preserved. Notably, the CHEMBL_4095823–PD-L1 complex displays a slight decrease in Rg over time, suggesting a minor compaction process, whereas the CHEMBL4089220–PD-L1 complex and the CHEMBL_2092784–PD-L1 complex maintain relatively stable yet slightly more variable profiles. Altogether, these results suggest that despite differences in local structural deviations captured by RMSD, the global folding and integrity of the systems remain conserved, with only subtle differences in dynamic compactness. Based on these results, further analyses were performed over the last 20 ns.

**Figure 4:**
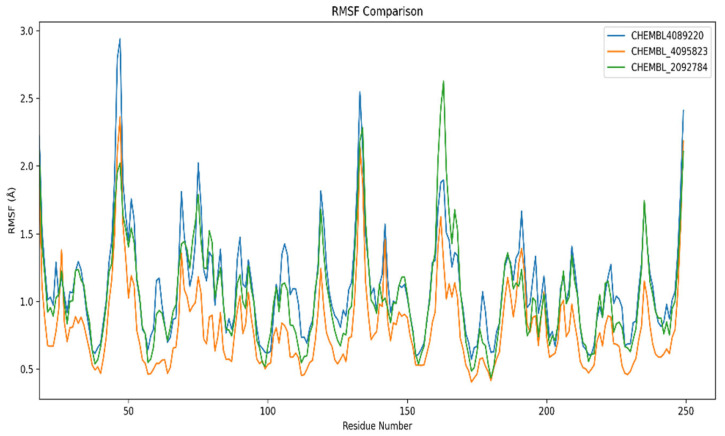
Root mean square fluctuation (RMSF) profiles of the protein backbone residues during the molecular dynamics simulations of the CHEMBL4089220–PD-L1, CHEMBL_4095823–PD-L1, and CHEMBL_2092784–PD-L1 complexes. RMSF values are reported in Å as a function of residue number, highlighting differences in local flexibility and conformational fluctuations among the analyzed systems.

The comparative RMSF analysis revealed moderate differences in local protein flexibility among the three ligand-bound systems (Figure 4). Overall, the complex containing CHEMBL4089220 exhibited the highest RMSF values across several regions, particularly around residues ~45–55, ~130–140, and near the C-terminal region (~245–250), indicating increased local fluctuations in these segments. In contrast, the CHEMBL_4095823 complex displayed the lowest RMSF values throughout most of the simulation, suggesting a comparatively more stable conformational behavior. The CHEMBL_2092784 system showed an intermediate fluctuation profile, although a marked RMSF peak was observed around residues ~160–170, corresponding to the highest local fluctuation among the analyzed systems. Despite these differences, all systems exhibited similar overall fluctuation patterns, indicating preservation of the global structural architecture during the MD simulation time.

#### 2.2.2. Binding Free Energy Calculations

**Figure 5:**
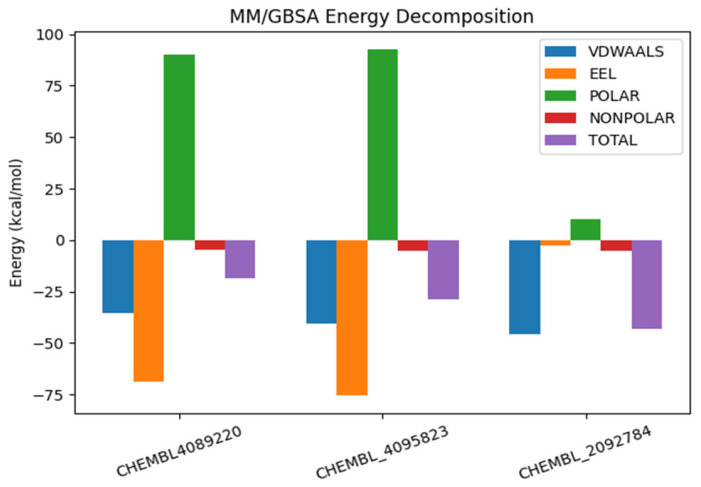
MM/GBSA binding free energy decomposition of the protein–ligand complexes for CHEMBL4089220, CHEMBL_4095823, and CHEMBL_2092784. Energy contributions are shown for van der Waals (VDWAALS), electrostatic (EEL), polar solvation (EGB), and nonpolar solvation (ESURF) components, along with the total binding free energy (ΔG_total). All values are reported in kcal/mol as the mean energies calculated over 100 trajectory frames.

The MM/GBSA binding free energy decomposition revealed distinct energetic contributions among the analyzed systems. The van der Waals (VDWAALS) interactions were consistently favorable across all compounds, with CHEMBL_2092784 showing the most negative contribution (−45.83 ± 0.26 kcal/mol), followed by CHEMBL_4095823 (−40.62 ± 0.32 kcal/mol) and CHEMBL4089220 (−35.60 ± 0.54 kcal/mol), indicating a stronger role of hydrophobic interactions in stabilizing the former system. Electrostatic contributions (EEL) were also favorable for CHEMBL4089220 (−68.56 ± 1.51 kcal/mol) and CHEMBL_4095823 (−75.61 ± 1.22 kcal/mol), whereas a markedly reduced electrostatic contribution was observed for CHEMBL_2092784 (−2.64 ± 0.34 kcal/mol). In all cases, these favorable interactions were partially offset by the polar solvation term (EGB), which was positive and particularly high for CHEMBL4089220 (90.31 ± 1.38 kcal/mol) and CHEMBL_4095823 (92.80 ± 1.07 kcal/mol) but notably lower for CHEMBL_2092784 (10.39 ± 0.44 kcal/mol). The nonpolar solvation contribution (ESURF) remained slightly favorable across all systems, with comparable values ranging from −4.61 to −5.24 kcal/mol (Figure 5).

As a result of these contributions, the total binding free energy (ΔG_total) differed significantly among the systems. CHEMBL_2092784 exhibited the most favorable binding affinity (−43.26 ± 0.39 kcal/mol), followed by CHEMBL_4095823 (−28.67 ± 0.37 kcal/mol) and CHEMBL4089220 (−18.45 ± 0.46 kcal/mol). These results indicate that the stronger binding observed for CHEMBL_2092784 is primarily driven by enhanced van der Waals interactions and a reduced desolvation penalty, while the other systems show greater compensation between electrostatic and polar solvation effects. The relatively low SEM values across all energy components further support the reliability and convergence of the simulations.

### 2.3. ADMET Analysis

**Figure 6:**
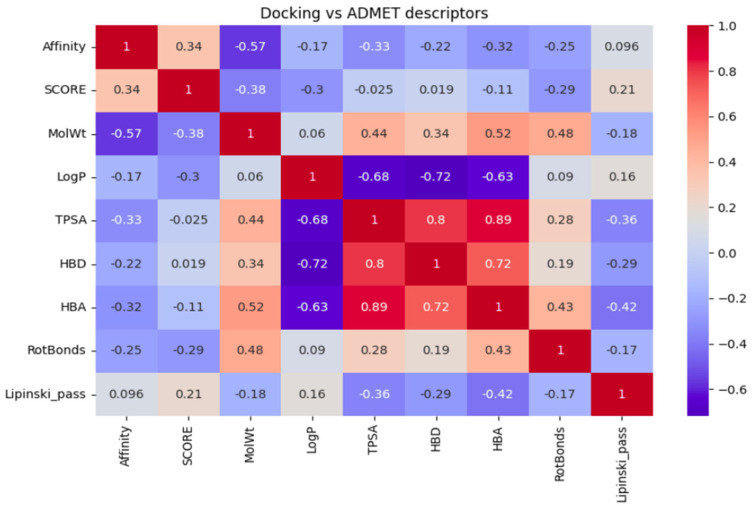
Molecular descriptor heatmap. Correlation matrix between descriptors used the following: affinity; energies of docking analysis; SCORE: similarity between cordycepin and other compounds of the database; MolWT: molecular weight; LogP: logarithm of the Octanol–Water Partition Coefficient; TPSA: topological polar surface area; HBD: hydrogen bond donor; HBA: hydrogen bond acceptor; RotBonds: rotatable bonds; Lipinski_pass: compliance with Lipinski’s rule.

ADMET profiling was performed to prioritize compounds whose physicochemical properties fall within the chemical space historically associated with CNS drugs, thereby increasing the probability of brain exposure. Particular emphasis was placed on descriptors strongly linked to blood–brain barrier permeability, including lipophilicity (logP, logD), topological polar surface area (TPSA), molecular weight, and hydrogen bonding capacity (HBD; HBA: hydrogen bond donor and hydrogen bond acceptor, respectively), as these parameters collectively influence passive diffusion across the BBB (Figure 6).

The analysis revealed that several top-ranked docking candidates also exhibited favorable pharmacokinetic descriptors, including moderate lipophilicity (logP = 2 to 4) [35], reduced topological polar surface area (TPSA = 40 Å to 90 Å) [22], molecular weight (<450–500 g/mol), HBD (low), and compliance with the drug-likeness criteria of Lipinski’s rule [36].

These features are commonly associated with an increased probability of brain exposure. Importantly, the integration of ADMET filters enabled the identification of compounds combining strong predicted binding affinity with properties supportive of CNS penetration, thereby reducing the likelihood of advancing molecules with suboptimal pharmacokinetic behavior. While BBB permeability is inherently multifactorial and cannot be inferred exclusively from physicochemical parameters, the applied framework served as an effective early-stage prioritization strategy to enrich the screened library with CNS-compatible candidates. The highest-ranked molecules were subsequently advanced to MPO analysis to further refine compound selection. These results provided the structural basis for subsequent MPO prioritization and ADMET profiling.

### 2.4. MPO Index

The MPO index was implemented as an integrative prioritization strategy to identify compounds displaying a balanced physicochemical profile suitable for CNS drug discovery. MPO frameworks are widely employed in early-stage drug discovery to enrich chemical libraries and guide compound selection beyond binding affinity alone.

In this study, the MPO approach incorporated descriptors such as topological polar surface area (TPSA), lipophilicity (logP), logBB, and drug-likeness criteria (Lipinski’s rule) to favor molecules with CNS-compatible characteristics. All descriptors were normalized prior to score integration to prevent parameter dominance and ensure a balanced contribution of each variable. Weights were assigned to reflect the relative pharmacological importance of each parameter in the context of glioblastoma drug discovery, prioritizing target engagement and CNS compatibility over general drug-likeness descriptors. Accordingly, the MPO index should be interpreted as a chemical prioritization tool designed to reduce the risk of advancing compounds with favorable predicted binding but inadequate pharmacokinetic potential (Table 4).

**Figure 7:**
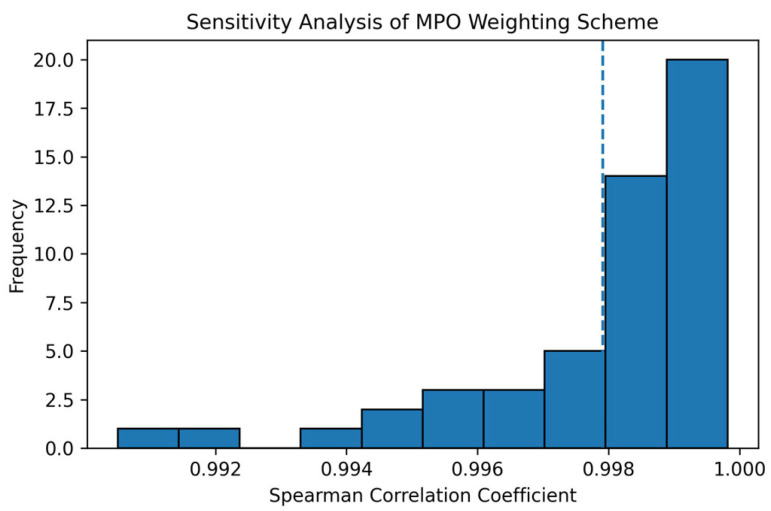
Sensitivity analysis of the MPO weighting scheme. Distribution of Spearman correlation coefficients obtained from the sensitivity analysis of the MPO weighting scheme. Random perturbations (±20%) were applied to the weighting factors across multiple iterations. The consistently high correlation values (mean ≈ 0.998) indicate that the compound ranking is highly robust and minimally affected by variations in the assigned weights. Dashed line indicates the average value of Spearman correlation coefficient near 0.998.

To evaluate the robustness of the multiparametric optimization (MPO) model, a sensitivity analysis was performed by introducing random perturbations (±20%) to the weighting scheme (Figure 7).

The resulting compound rankings remained highly consistent with the original prioritization, yielding an average Spearman correlation coefficient of 0.998 and a minimum value of 0.991 across all iterations. Furthermore, the overlap among the top five ranked compounds remained high, with an average agreement of 91.2%. These findings indicate that the MPO-based ranking is highly stable and not significantly influenced by variations in the weighting parameters, supporting the reliability of the prioritization strategy.

## 3. Discussion

The present study investigated the potential of nucleoside-derived compounds as modulators of PD-L1 using an integrated computational workflow that combines molecular docking, MD simulations, physicochemical descriptor analysis (ADME), and MPO analysis [37]. This strategy allowed the identification of candidate molecules that not only display favorable predicted binding affinity but also exhibit physicochemical characteristics compatible with drug-like behavior, particularly in the context of CNS drug discovery.

The initial molecular docking screening identified a subset of compounds with predicted binding affinities comparable to those reported for previously described small-molecule inhibitors of PD-L1. Although docking scores should be interpreted cautiously and do not constitute direct evidence of biological activity, the results suggest that nucleoside-related scaffolds may be capable of establishing stabilizing interactions within the PD-L1 homodimer binding interface [38].

Structural studies have shown that effective PD-L1 inhibitors frequently bind within a hydrophobic pocket formed at the PD-L1 dimer interface, where residues such as Tyr56, Met115, Ala121, and Tyr123 contribute to ligand stabilization [33]. The predicted binding poses obtained in the present study are consistent with this structural model and support the plausibility of nucleoside-based ligands interacting with this region of the protein (Table 3).

An important observation emerging from the dataset is the presence of the known PD-L1 inhibitor BMS-1 [39] among the screened molecules. Interestingly, this compound ranked among the highest-scoring candidates according to both docking affinity and the integrated MPO score.

The identification of a previously reported PD-L1 ligand among the top-ranked compounds provides indirect support for the validity of the docking protocol and the selected binding region presented in this work. While such observations do not constitute experimental validation, they suggest that the computational workflow applied in this study is capable of capturing structural features associated with known PD-L1 binding chemotypes [40].

Despite these encouraging docking results, it is well established that docking-based ranking alone is insufficient for reliable compound prioritization [41,42]. Molecules with favorable docking scores may still possess physicochemical properties incompatible with adequate pharmacokinetics or bioavailability [37]. This limitation is particularly relevant for therapeutic strategies targeting brain tumors such as glioblastoma, where the ability of small molecules to cross the BBB is a critical factor influencing therapeutic efficacy [43]. For this reason, additional descriptors related to drug-likeness and potential brain exposure were evaluated for all compounds.

The observed energy decomposition in the MD simulation study suggests that ligand binding is primarily driven by van der Waals interactions, highlighting the importance of hydrophobic contacts and steric complementarity within the binding site. These additional MD simulations were performed to further validate the docking results, specifically to assess whether the top-ranked compounds could maintain their predicted binding poses under dynamic conditions. For this purpose, three of the highest-ranked ligands were selected and subjected to molecular dynamics simulations, allowing evaluation of the stability of the protein–ligand complexes and whether the ligands remain within the predicted binding site.

The results demonstrate that the selected compounds preserve their binding mode throughout the simulations, supporting the reliability of the docking predictions. Although electrostatic interactions contribute favorably, their effect is largely compensated by the polar solvation penalty, a common feature in MM/GBSA analyses of protein–ligand systems. This balance underscores the critical role of solvent effects in modulating binding affinity.

The consistently negative ESURF values further support the contribution of hydrophobic interactions to complex stabilization. Variations in ΔG_total between systems likely arise from differences in conformational sampling and dynamic interaction networks, as also suggested by the RMSD and radius of gyration analyses. Systems exhibiting more favorable ΔG_total values are indicative of stronger and more stable binding modes.

In agreement with these observations, RMSF analysis revealed moderate differences in local flexibility among the analyzed systems. CHEMBL4089220–PD-L1 displayed higher fluctuations in several regions, whereas CHEMBL_4095823–PD-L1 showed comparatively lower RMSF values, suggesting increased structural stability during the simulations. In contrast, CHEMBL_2092784–PD-L1 exhibited localized flexibility peaks, indicating distinct dynamic behavior despite preservation of the overall protein architecture.

Overall, these results emphasize a binding mechanism dominated by dispersion interactions, with electrostatics and solvation effects fine-tuning the overall affinity. The low SEM values further indicate that the simulations achieved adequate convergence, lending confidence to the reliability of the calculated binding free energies.

The analysis of physicochemical parameters of compounds revealed that several of the top-ranked candidates display properties commonly associated with drug-like molecules; in particular, several compounds exhibited molecular weights within ranges typical for small-molecule drugs and moderate lipophilicity values compatible with membrane permeability (Supplementary File S1). In addition, several molecules presented topological polar surface area (TPSA) values that fall within ranges frequently associated with an increased probability of CNS exposure [44]. Although these parameters alone cannot guarantee BBB penetration, they provide useful preliminary indicators for prioritizing compounds in early-stage drug discovery (e.g., integrated SAR/QSPR models and physicochemical descriptors are widely used for initial screening despite their predictive limitations) [45].

To further refine candidate selection, an MPO score was calculated by integrating docking affinity with key physicochemical descriptors, including lipophilicity, polar surface area, molecular weight, and predicted BBB permeability (Supplementary File S2). The comparison between docking-based ranking and MPO-based prioritization revealed notable differences in compound ordering. Several molecules exhibiting strong docking scores were deprioritized due to unfavorable physicochemical characteristics, whereas other compounds with slightly lower docking affinities achieved higher MPO scores because of a more balanced combination of structural and pharmacokinetic properties (Table 5). These findings highlight the importance of integrating multiple descriptors during early-stage virtual screening to reduce the selection of false-positive hits that may arise when docking scores are considered in isolation.

Another noteworthy observation concerns the structural characteristics of the compounds with the highest MPO scores. Several of these molecules retain structural elements typical of nucleoside scaffolds while incorporating modifications that increase lipophilicity and reduce overall polarity (Figure 1). Such structural adjustments are consistent with medicinal chemistry strategies commonly employed to improve the pharmacokinetic properties of nucleoside analogs [46]. Natural nucleosides such as Cordycepin are known to display diverse biological activities [47] but often exhibit limited membrane permeability due to their relatively high polarity and dependence on nucleoside transporters [48]. The identification of modified nucleoside-like structures among the highest-ranked candidates therefore suggests that this scaffold may represent a promising starting point for the design of PD-L1 modulators with improved drug-like characteristics.

Despite the promising computational observations reported in this work, several limitations must be acknowledged [49]. Molecular docking provides only an approximate representation of ligand–protein interactions and does not account for the dynamic conformational behavior of proteins or solvent effects [50,51]. Furthermore, the ADMET properties evaluated in this study rely on predictive models and should therefore be interpreted as preliminary indicators rather than definitive pharmacokinetic measurements [52,53]. Experimental validation through biochemical binding assays, cellular studies, and eventually in vivo models will be necessary to determine whether the identified compounds can effectively modulate PD-L1 signaling in biological systems.

## 4. Methods and Materials

### 4.1. Docking Analysis

#### 4.1.1. Ligand Library Preparation

A chemical library comprising 400 compounds was obtained from the Swiss Similarity database [54]. Molecular structures were retrieved as SMILES strings and converted into three-dimensional structures. Ligands were subsequently transformed into SDF format and prepared for docking through protonation state assignment and geometry optimization. PDBQT files were generated using Open Babel 3.1.0 [55] via command-line execution in a Linux environment.

#### 4.1.2. Protein Preparation

The receptor structure was obtained from the Protein Data Bank (PDB ID: 8OR1). Protein preparation involved the removal of crystallographic water molecules, the addition of polar hydrogens, and the assignment of Kollman charges to ensure compatibility with the docking software.

The docking grid was centered on the native ligand binding site to accurately represent the biologically relevant interaction region. Center coordinates were defined as X = −34, Y = 39, and Z = −19, and X = 19, Y = 18, and Z = −25 for size.

#### 4.1.3. Molecular Docking

Docking simulations were performed using AutoDock Vina 1.5.7 [56], employing an exhaustiveness parameter of 32. Compounds were ranked according to their predicted binding affinity values (kcal/mol), with more negative energies interpreted as stronger predicted binding.

#### 4.1.4. Docking Validation

To validate the docking protocol, a redocking procedure was conducted using the cocrystallized ligand VYC from the original PDB structure for PDL1. To confirm this result, two other known inhibitors were used, as reported in Table 1. Docking accuracy was assessed by calculating the RMSD between the experimental and predicted poses.

### 4.2. Molecular Dynamics Simulation with MMGBSA Approach

Receptor–ligand complexes were prepared using the Antechamber and TLeap modules implemented in the AMBER22 package [57]. The systems were described using the ff14SB force field for the protein, while ligand parameters were assigned using the General Amber Force Field (GAFF) with AM1-BCC atomic charges [58,59]. Each system was solvated with the TIP3P water model in a truncated octahedral box extending 12 Å from the solute and treated under periodic boundary conditions (PBCs). Counterions were added to neutralize the system, and additional Na^+^ and Cl^−^ ions were included to achieve a physiological salt concentration of 0.15 M.

Energy minimization was carried out using 5000 steps of steepest descent followed by 4000 steps of conjugate gradient. The system was then gradually heated from 0 to 310 K over 200 ps under an NVT ensemble using a Berendsen thermostat algorithm [60]. Density equilibration was subsequently performed for 200 ps under an NPT ensemble, employing a Langevin thermostat and a Berendsen barostat [61]. Production MD simulations were conducted for 100 ns at 310 K under NPT conditions using a 2 fs time step.

Long-range electrostatic interactions were treated using the Particle Mesh Ewald (PME) method, while a 10 Å cutoff was applied for nonbonded interactions [62]. All covalent bonds involving hydrogen atoms were constrained using the SHAKE algorithm [63]. Trajectory analyses, including RMSD, Rg, RMSF, and clustering, were performed using the CPPTRAJ module.

#### Binding Free Energy Calculations

Binding free energies (ΔG_bind) were estimated using the Molecular Mechanics with Generalized Born Surface Area (MM/GBSA) approach [64], based on 100 snapshots extracted from the last 20 ns of the equilibrated trajectories. Calculations were performed using an implicit solvent model, as previously described [65,66].

### 4.3. ADMET Prediction

ADMET (Absorption, Distribution, Metabolism, Excretion, and Toxicity) properties were evaluated to support the early prioritization of candidate molecules for glioblastoma therapy, with particular emphasis on descriptors associated with blood–brain barrier (BBB) permeability.

Physicochemical parameters, including lipophilicity (logP), topological polar surface area (TPSA), and other drug-likeness criteria, were computed using atomic fragments with RDKIT software following Crippen’s method [67]. These descriptors were selected due to their empirical association with central nervous system exposure.

Given that BBB permeability is a multifactorial process influenced by transporter activity, plasma protein binding, metabolic stability, and efflux mechanisms such as P-glycoprotein, the ADMET framework was implemented as a chemical enrichment strategy rather than a definitive predictor of brain penetration. Compounds demonstrating favorable ADMET profiles were prioritized for subsequent MPO-based ranking.

### 4.4. MPO Scoring

A MPO framework was implemented to integrate binding affinity and ADMET-related descriptors into a single prioritization metric. The MPO score was designed to balance target engagement with physicochemical and permeability properties relevant to central nervous system drug discovery.

Binding affinity values obtained from molecular docking were transformed to a positive scale and normalized using min–max scaling [37]. Predicted blood–brain barrier permeability (logBB), obtained from the pkCSM platform [68], was likewise normalized to ensure comparability across descriptors, facilitating integration within the multiparameter optimization framework.

Drug likeness was evaluated according to Lipinski’s rule, and a continuous Lipinski compliance score was defined as follows: Lipinski score = 1 − (number of violations/4). Additional physicochemical descriptors were incorporated as categorical scoring functions reflecting empirically favorable ranges for CNS-active compounds. These included molecular weight (MW), lipophilicity (LogP), and TPSA. All descriptors were normalized using min–max scaling to ensure comparability across parameters and to prevent dominance of variables with larger numerical ranges. Each descriptor was assigned a score between 0 and 1 based on predefined optimal intervals associated with CNS exposure.

The final MPO score was calculated as a weighted linear combination of normalized and scaled parameters:

MPO_score = 0.40 × Affinity_norm + 0.20 × logBB_norm + 0.15 × Lipinski_score + 0.10 × TPSA_score + 0.10 × LogP_score + 0.05 × MW_score(1)

Weights were assigned according to pharmacological rationale (Table 4). Binding affinity (free energy values) was prioritized as the primary driver of target engagement, while BBB permeability was emphasized due to its critical relevance in glioblastoma therapy. The remaining parameters were incorporated to ensure balanced physicochemical properties compatible with drug-like behavior and CNS exposure. Sensitivity analysis will study whether the MPO ranking is stable or not, with negligible variation across different weighting schemes (±20), indicating that compound prioritization is not driven by arbitrary parameter selection.

The MPO score ranges from 0 to 1, with higher values indicating compounds that simultaneously exhibit strong predicted binding, favorable permeability, and optimal physicochemical profiles.

## 5. Conclusions

In summary, the integrated computational workflow applied in this study enabled the identification of nucleoside-derived molecules with predicted affinity for PD-L1 and physicochemical characteristics compatible with further optimization as drug candidates. The results support the potential of nucleoside-based scaffolds as starting points for the development of small-molecule modulators targeting immune checkpoint pathways in glioblastoma. Future studies combining MD simulations, structure-guided optimization, and experimental validation will be necessary to further explore the therapeutic potential of these compounds.

## Figures and Tables

**Table 1:** Experimental antitumor evidence for cordycepin inhibitors and PD-L1 inhibitors.

Compound and Dose	Study Model	Conclusion
Cordycepin (10–200 µM)	Cell lines: U251, T98G	It inhibits proliferation and migration and reduces PD-L1 expression by regulating NF-κB/STAT1 [27].
Cordycepin and Temozolomide (50, 100, 200, 400 µM), respectively	Cell lines: rat C6 as well as human LN18, T98G, U87, and U251 and in vivo mice model	Cordycepin alone and in combination with temozolomide inhibited tumor growth, migration, and metastasis, as well as the induction of apoptosis and cell cycle arrest in vitro. In vivo, this combination decreased tumor volume and prolonged survival time, with AMPK activation and AKT suppression being among the most important mechanisms [28].
Cordycepin (80 µM) and doxorubicin (1 µM)	Cell lines: LN-229, U251 and T98G cells	The combination can significantly inhibit the viability, migration, and proliferation capacity of U251 and T98G cells [29].
BMS-1166	In vitro by coculture of glioma cells with microglia; in vivo rat glioma model	Blocking the PD-1/PD-L1 axis suppresses M2-type polarization of microglia and reduces glioblastoma invasion [30].
BMS-202 (10 µM)	Cell lines: U251, LN229 and HEB	BMS-202 inhibits the proliferation of glioblastoma multiforme cells by blocking cell migration and invasion. It also reduces PD-L1 expression on the cell surface and disrupts the PD-L1-AKT-BCAT1 axis independently of mTOR signaling [31].

**Table 2:** Redocking procedure for the main PD-L1 crystals.

	Crystals of PD-L1		
	8OR1	5J89	6NM8
RMSD (Å)	1.96	0.86	1.78
Inhibitor	VYC ^1^	6GX ^2^	KSD ^3^
kcal/mol	−10.9	−11.0	−10.0
H-Bond Interactions	Asp122, Tyr123, Lys124,	Tyr56, Gln66, Tyr123	Asp122, Lys124

^1^: PubChem: 168433094 (https://pubchem.ncbi.nlm.nih.gov/compound/168433094), ^2^: PubChem 117951478, ^3^: PubChem 117942007.

**Table 3:** Compounds with the predicted strongest interactions with PD-L1.

Compound	Affinity ^1^ (kcal/mol)	2D Structure
VYC ^2^	−10.9	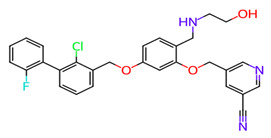
CHEMBL4077793	−10.8	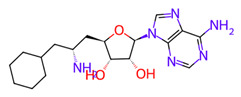
CHEMBL4089220	−10.4	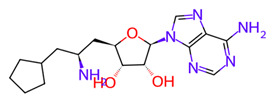
BMS-1 ^3^	−10.3	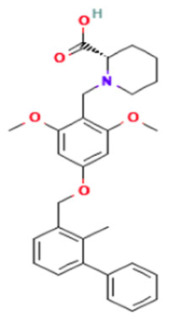
CHEMBL4095823	−10.2	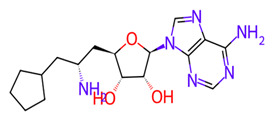
CHEMBL4074394	−9.8	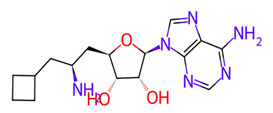
CHEMBL2092784	−9.7	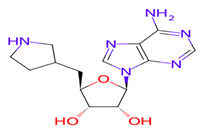
CHEMBL4556165	−9.6	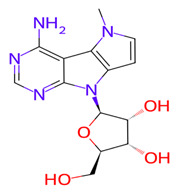
CHEMBL4095401	−9.6	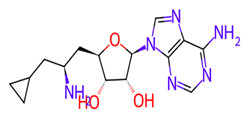
CHEMBL4085649	−9.6	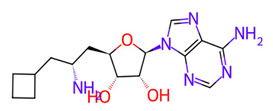

^1^ Free energy values calculated from docking analysis. ^2^ PubChem: 168433094 (https://pubchem.ncbi.nlm.nih.gov/compound/168433094). ^3^ PubChem: 91663303.

**Table 4:** Parameters used to construct the MPO Index.

Parameter	Weights	Description
Affinity_norm	0.40	Docking energies. Affinity_norm = (Affinity_max − Affinity_compound)/(Affinity_max − Affinity_min)
LogBB	0.20	logBB = log10(Cbrain/Cblood) ^1^
Lipinski_score	0.15	Lipinski_score = rules compliance/4 = 1.0
TPSA_score	0.10	TPSA_score = (TPSA_max − TPSA_compound)/(TPSA_max − TPSA_min)
LogP_score	0.10	LogP_score = (LogP_compound − LogP_min)/(LogP_max − LogP_min)
MW_score	0.05	MW_score = (MW_max − MW_compound)/(MW_max − MW_min)

^1^ Calculation of the pkCSM platform.

**Table 5:** Interaction of the ligands reported with the highest MPO_index and known ligands.

Compound	Binding Energy (kcal/mol)	MPO_Index	Interacting Residues			
			Hydrogen Bonds	Hydrophobic Interactions	Pi Interactions	2D Interactions
BMS-1	−10.3	0.81	Met115	Met115A, Ala121A, Tyr123A, Tyr123B, Asp122A,	Tyr56B	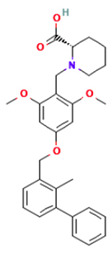
VYC	−10.9	0.81	Asp122A, Tyr123A, Lys124A	Ala121A, Met115A, Tyr123B, Asp122A	Tyr56B, Tyr123B	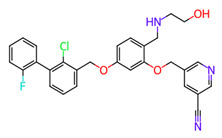
CHEMBL 4077793	−10.8	0.84	Ile54B, Met115B, Ile116B, Asp122B	Tyr56B, Met115B, Asp122A	Tyr123B	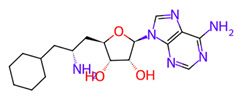
CHEMBL 4089220	−10.4	0.81	Cys114A, Met115B, Ile116B	Tyr56B, Met115B, Asp122A	Tyr123B	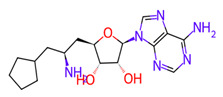
CHEMBL 4095823	−10.2	0.78	Cys114A, Ile116B	Ala121A, Asp122A, Met115A, Tyr56B	Tyr123B	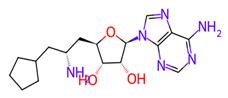
CHEMBL 4074394	−9.8	0.74	Cys114A, Ile116B	Met115A, Tyr56B	Tyr123B	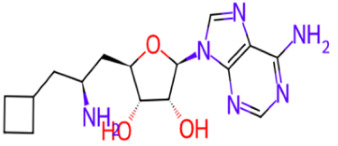
CHEMBL 2092784	−9.7	0.73	Glu58A, Met115A, Met115B	Met115B, Ala121A	-	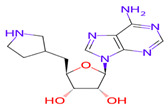
CHEMBL 1222699	−9.3	0.73	Ile54B, Cys114A	Met115B	-	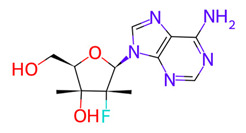
CHEMBL 4556165	−9.6	0.72	Ile54B, Cys114A	Met115B	-	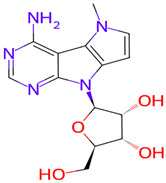
Cordycepin	−7.7	0.56	Asp122B, Ile116B	Met115A, Ala121A	-	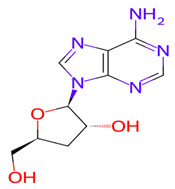

## Data Availability

The original contributions presented in this study are included in the article. Further inquiries can be directed to the corresponding authors.

## Supplementary Materials

The following supporting information can be downloaded at: https://www.mdpi.com/article/10.3390/ijms27115024/s1.

## Abbreviations

The following abbreviations are used in this manuscript:

MPO	Multiparameter optimization
PD-L1	Programmed death ligand-1
