# Supplementary material for: Fungal Cordyceps Nucleosides and Analogs as Potential Anti-Glioblastoma PD-L1 Inhibitors: An In Silico Multiparameter Optimization (MPO) Design
Source: Int J Mol Sci. 2026 Jun 2;27(11):5024. doi: 10.3390/ijms27115024 (PMC13256099; doi:10.3390/ijms27115024)
Supplement: Supplementary file 1 [file ijms-27-05024-s001.zip › Suplement 1.pdf]

Redocking validation

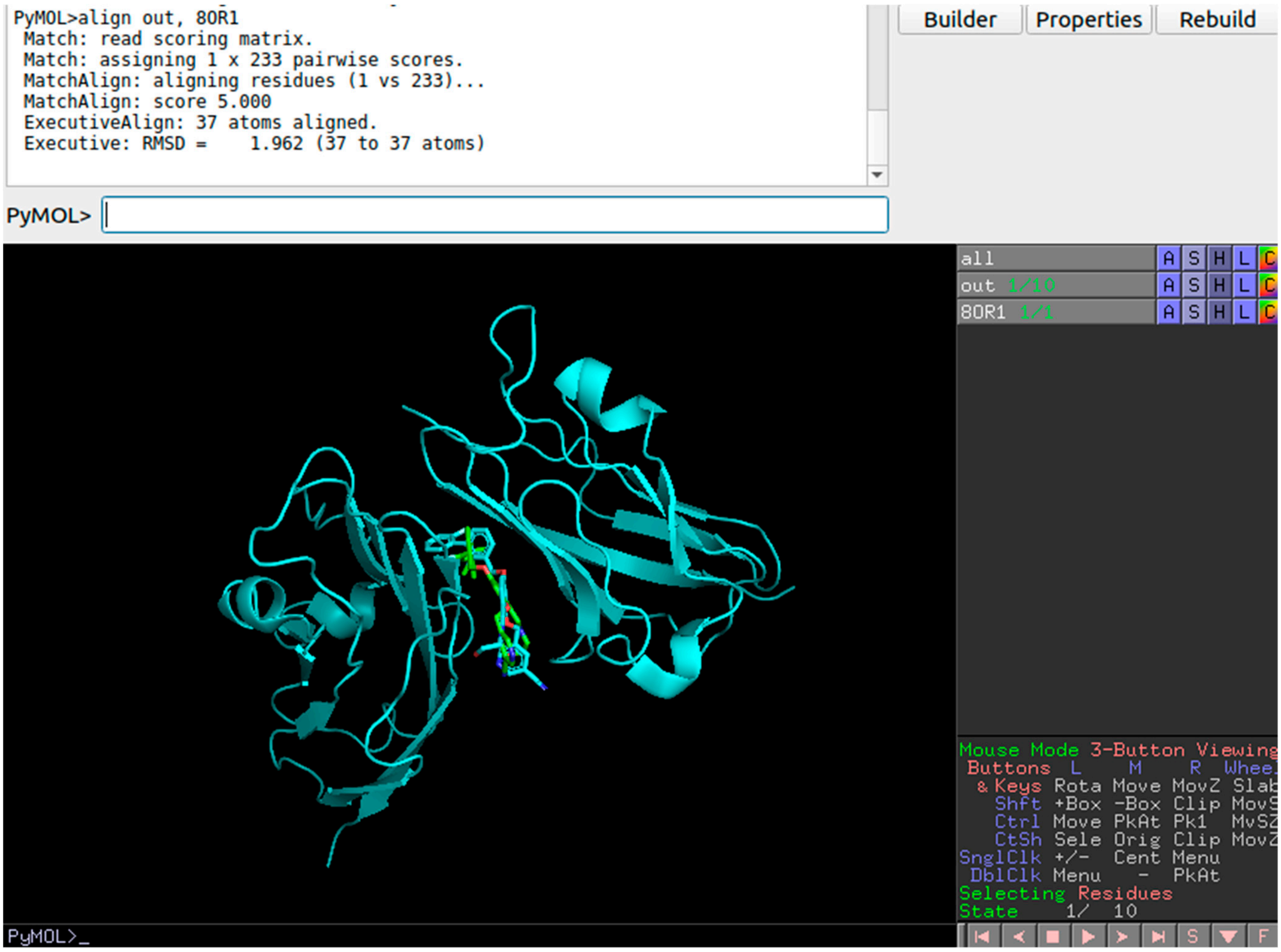

Redocking validation comparing poses of VYC ligand. “Out”pose is VINA result. 8OR1 is PDB Crystal with ligand

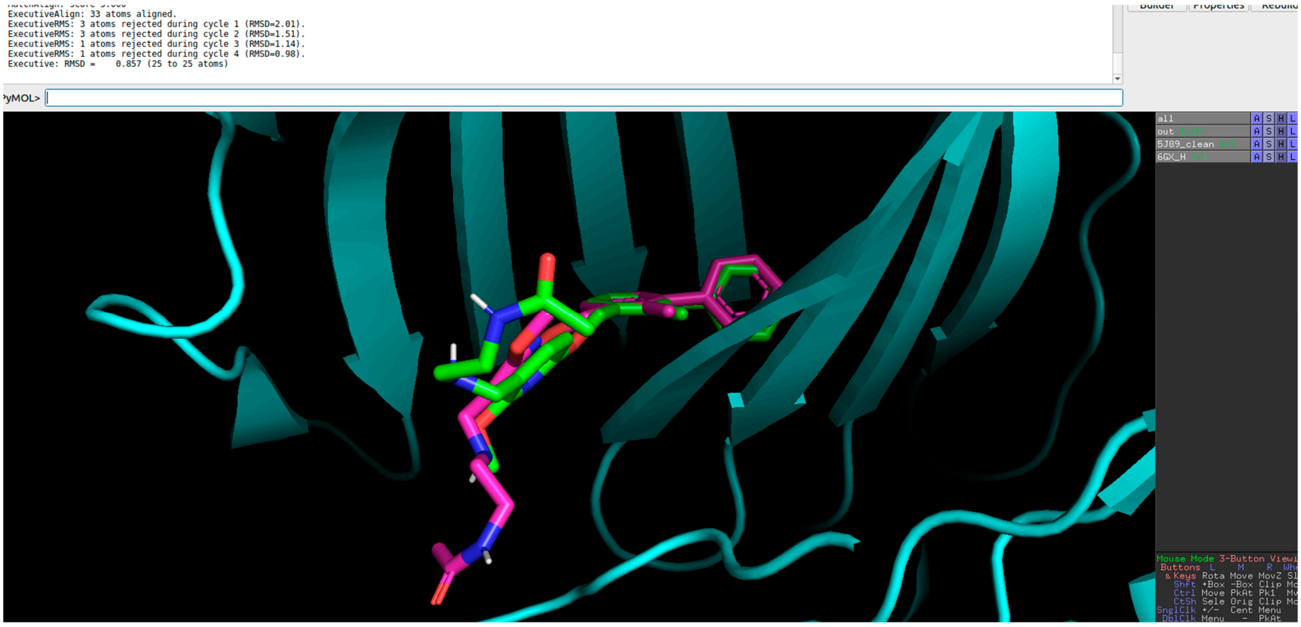

5J89 Redocking validation comparing poses of 6GX ligand

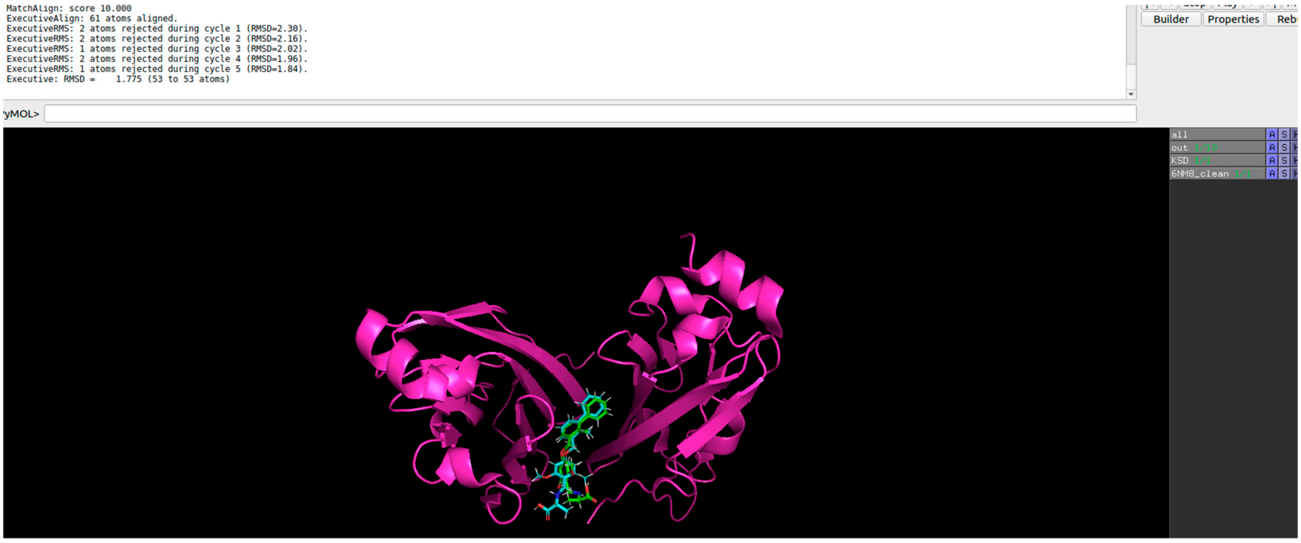

6NM8 Redocking validation comparing poses of KSD ligand
